# Functional Analysis of Human Hub Proteins and Their Interactors Involved in the Intrinsic Disorder-Enriched Interactions

**DOI:** 10.3390/ijms18122761

**Published:** 2017-12-19

**Authors:** Gang Hu, Zhonghua Wu, Vladimir N. Uversky, Lukasz Kurgan

**Affiliations:** 1 School of Mathematical Sciences and LPMC, Nankai University, 300071 Tianjin, China; huggs@nankai.edu.cn (G.H.); wuzhh@nankai.edu.cn (Z.W.); 2 Department of Molecular Medicine and Byrd Alzheimer’s Research Institute, Morsani College of Medicine, University of South Florida, Tampa, FL 33612, USA; 3 Laboratory of New Methods in Biology, Institute for Biological Instrumentation of the Russian Academy of Sciences, Pushchino, Moscow region 142290, Russia; 4 Department of Computer Science, Virginia Commonwealth University, Richmond, VA 23284, USA

**Keywords:** intrinsic disorder, protein-protein interactions, hub proteins, intrinsically disordered proteins, human proteome

## Abstract

Some of the intrinsically disordered proteins and protein regions are promiscuous interactors that are involved in one-to-many and many-to-one binding. Several studies have analyzed enrichment of intrinsic disorder among the promiscuous hub proteins. We extended these works by providing a detailed functional characterization of the disorder-enriched hub protein-protein interactions (PPIs), including both hubs and their interactors, and by analyzing their enrichment among disease-associated proteins. We focused on the human interactome, given its high degree of completeness and relevance to the analysis of the disease-linked proteins. We quantified and investigated numerous functional and structural characteristics of the disorder-enriched hub PPIs, including protein binding, structural stability, evolutionary conservation, several categories of functional sites, and presence of over twenty types of posttranslational modifications (PTMs). We showed that the disorder-enriched hub PPIs have a significantly enlarged number of disordered protein binding regions and long intrinsically disordered regions. They also include high numbers of targeting, catalytic, and many types of PTM sites. We empirically demonstrated that these hub PPIs are significantly enriched among 11 out of 18 considered classes of human diseases that are associated with at least 100 human proteins. Finally, we also illustrated how over a dozen specific human hubs utilize intrinsic disorder for their promiscuous PPIs.

## 1. Introduction

Although proteins are engaged in virtually all aspects of cellular functionality, they rarely act alone, being normally involved in multiple interactions (i.e., specific physical contacts that have functional consequences) with other proteins, peptides, nucleic acids, polysaccharides, various small molecules, lipids, etc. This is reflected in the fact that proteins in a cell are traditionally described in a form of specific pathways that represent a series of actions among molecules leading to a particular change in a cell, e.g., generation of a certain product. Therefore, mapping of protein-protein interactions (PPIs) represents an important step towards the better understanding of the complex molecular relationships in living systems [1]. In fact, the resulting intricate PPI networks, or interactomes, are considered now as a gateway into systems biology, since they represent an important means to understand how biochemical and biophysical characteristics of proteins can be integrated into biological systems [2]. Interactomes are different from canonical pathways, being typically more complex and providing massive complementary information that reveals a multitude of links (or interactions) of each of the proteins included in a given network.

Interactomes have complex topologies, where proteins are depicted as nodes and PPIs are shown as links or connections between these nodes. Complex networks found in many diverse systems are typically assumed to be random [3]. Given a random placement of links in the random networks, most nodes have approximately the same number of links [3,4]. In other words, in a completely random network, the connectivity of nodes (also known as degree) follows a Poisson distribution with a bell shape. This means that it is difficult to find nodes that have significantly more or fewer links than the network average values [4]. On the other hand, PPI networks inside cells are classified as “scale-free” or “small-world” networks, as systematized in [5]. Other examples of such networks include the author-collaboration networks, the airline routes, the World Wide Web, the metabolic networks, and the protein domain networks. Degree distribution in these scale-free networks follows a power law [6], suggesting the presence of nodes with a degree that greatly exceeds the network average value. In other words, although in scale-free networks most nodes have just a few links, there are some popular nodes (hubs) with a very large number of connections to other nodes (they can have hundreds, thousands or even millions of links), as well as there are some nodes (ends) connected only to one other node [4,5,7]. Therefore, the geometry of scale-free networks combines the local clustering of connections characteristic of regular networks with occasional long-range connections between clusters, as can be expected to occur in random networks [7,8].

While there is no established consensus in the literature on how to define hub proteins, many studies define them as proteins with larger degrees in the PPI networks, when compared to the non-hub proteins. For instance, one of the early studies by Han et al. [9] defined hubs as proteins with degrees >5, Haynes et al. [10] decided to increase the cut-off to over 10, while Batada et al. [11] defined hubs as the top 5% of proteins with the highest degrees. In more recent studies, Aragues et al. [12] specified hubs as proteins with degrees >20, and Jin et al. [13] as the top 20% of proteins with the highest degrees. With respect to timing of PPIs in networks, some hubs have multiple, simultaneous interactions (“party hubs”), forming scaffolds that enable the assembly of functional modules [9], whereas others hubs have multiple sequential interactions (“date hubs”) [9] and are likely to play a role in connecting biological modules to each other [14]. Irrespective of all these issues and considerations, hubs are invariantly characterized by an intrinsic ability to be engaged in multiple interactions with numerous (often unrelated) partners. This ability to utilize the “one-to-many” interaction mode moves hubs from the comfort of the traditional lock-and-key model, if each given PPI would be considered as one function. Such binding promiscuity also raises an important question on structural determinants of hubness.

The “one protein-one structure-one function” paradigm has morphed in recent years, as it is becoming progressively more evident that many proteins are biologically active despite the lack of a unique 3D-structure either entirely or in their significant parts [15,16,17,18,19,20,21]. These intrinsically disordered proteins (IDPs) and intrinsically disordered protein regions (IDPRs) represent a unique and important extension of the protein kingdom, being abundantly present in any given proteome, as evidenced by several systematic bioinformatics analyses [22,23,24,25,26]. Functions of IDPs/IDPRs are complementary to functions of ordered proteins and domains [18,27,28,29]. Furthermore, dysfunction and dysregulation of many IDPs and hybrid proteins (i.e., that have ordered domains and long IDPRs) results in protein misfolding and aggregation, loss of normal function, and gain of toxic function [30,31,32], which are associated with the development of various diseases [31,33,34,35,36].

Among the unique disorder-specific functional features of IDPs and IDPRs is their exceptional binding promiscuity, where one protein or region is able to bind to multiple partners [37]. Obviously, the classical molecular recognition mechanisms cannot explain the ability of IDPs/IDPRs to bind to multiple partners [38]. In fact, neither the lock-and-key [39] nor original induced-fit [40] mechanisms that were developed to describe recognition behavior of ordered proteins give a reasonable description of this multi-binding capability. Furthermore, some IDPs/IDPRs were shown to adopt different structures upon binding to different partners [15,41,42,43,44,45,46], thereby playing a number of crucial roles in mediating PPIs [10,15,37,41,42,43,44,45,46,47,48,49,50,51,52,53,54,55,56,57,58,59].

Based on these observations and on the fact that IDPs/IDPRs are commonly involved in one-to-many and in many-to-one binding [15,60], it was proposed that molecular recognition via disorder-to-order transitions upon binding would be a reasonable mechanism of binding by hub proteins [61]. The overall importance of intrinsic disorder for functions of hub proteins was analyzed in several recent bioinformatics studies [10,47,48,49,50,59]. Although disorder is more clearly associated with date hubs than with party hubs [49,59], some protein complexes clearly use long IDPRs as scaffolds to assemble groups of interacting proteins [51,52,53,54,55,56,57,58,62].

Although several previous studies were specifically dedicated to the analysis of the enrichment of disorder in hubs [10,47,48,49,59], the functional peculiarities of PPIs involving hubs (hub PPIs) remains mostly unexplored. The goal of this study is to fill this gap and to provide detailed functional characterization of hub PPIs. We are the first to analyze abundance and functions of disorder for hub PPIs for both hubs and their interactors. We also investigate whether disease associated proteins are abundant among the proteins involved in the disorder-enriched hub PPIs. We focus our analysis on the human proteome given the relatively high degree of completeness of its PPI network [63] and relevance to the analysis of the disease-associated proteins.

## 2. Results and Discussion

### 2.1. Intrinsic Disorder in Hub Protein-Protein Interactions

**Figure 1:**
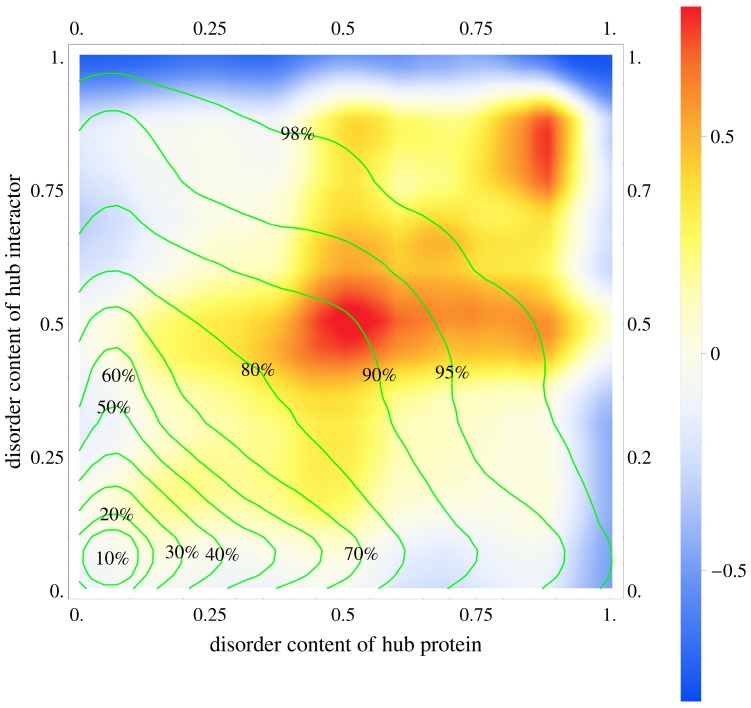
Enrichment in intrinsic disorder of human hub proteins and their interactors. The *x*- and *y*-axis show the amount of disorder content of the hubs and hub interactors, respectively. Each protein-protein interaction (PPI) is mapped into this two-dimensional plane and the density of these hub-interactor pairs is represented by green isolines. For instance, 40% of these pairs occupy the lower left corner where the disorder content of both hubs and interactors is below 0.25. The density was modelled with the Epanechnikov kernel function using Mathematica software. Next, we simulated a randomized PPI network that follows the same distribution of node density, i.e., we randomly assigned interactions between the human proteins to maintain the same density profile as in the true PPI network. Coloring of the inside of the two-dimensional plane reflects a relative ratio between the density of true (*d*_n_) and randomized (*d*_r_) interactions in the PPI networks calculated as [*d*_n_(x,y)-*d*_r_(x,y)]/*d*_r_(x,y). The color scale given on the right defines values of the ratio, e.g., orange corresponds to PPIs which are 0.5 times more frequent in the true PPI network compared to the random network.

Our aim was to annotate and functionally/structurally characterize human proteins involved in the hub PPIs that have higher than expected disorder content. To annotate these proteins, we compared distributions of the disorder content in the human hubs and their interactors between true and random human PPI networks. We utilized the random network to quantify the expected levels of disorder content. We defined the random network by generating PPIs between randomly chosen pairs of human proteins such that the number of interactions and the node densities are the same as in the true PPI network. Figure 1 compares the distribution of the PPIs in the two-dimensional space defined by the disorder content of the hubs (*x*-axis) and their interactors (*y*-axis), where the coloring represents a relative ratio between the density of the true and randomized interactions in the PPI networks. Using the color scale defined in Figure 1, the orange (light blue) corresponds to PPIs which are 0.5 times more (less) frequent in the true PPI network compared to the random network. The green isolines define the density of PPIs in that space.

The large rectangular region colored from orange to red in the top right quadrant of Figure 1 reveals a collection of about 3% of PPIs that have substantially higher than expected density in the two-dimensional intrinsic disorder space. The relative ratio of density is >0.5 in that region, which means that the density of the true PPIs that have the corresponding levels of intrinsic disorder in the hub proteins and its interactors is at least 50% higher than expected. More precisely, among the 141,346 PPIs that involve hubs (which we call “hub PPIs”), we found 4071 hub PPIs that have density enriched by at least 50% vs. 137,275 of the remaining hub PPIs (enrichment <50%). We note that the 141,346 hub PPIs are a subset of all 151,461 PPIs since they exclude interactions between non-hub proteins. There are 481 hubs and 1580 interactors that are involved in the 4071 hub PPIs. In agreement with the prior works that suggest that hubs have higher than expected amounts of the intrinsic disorder, the proteins that have substantially higher than expected density in the two-dimensional disorder space are characterized by large amounts of disorder, between 50% and 90%. Our analysis suggests that only a relatively small fraction of all hubs (481 out of 2866 = 16.8%) are in this set, while the remaining majority of hubs participates in hub PPIs that do not have higher than expected density; the latter set of hubs corresponds to hub PPIs that are located in the regions colored blue, white, and light yellow in Figure 1. Interestingly, the same is also true for the interactors of hubs. They follow a similar pattern of inclusion of only a relatively small fraction of proteins (1580 out of 13,963 = 11.3%) with substantially higher than expected density in the two-dimensional intrinsic disorder space. Like in the case of the hubs, these interactors are also characterized by large values of the disorder content. Moreover, our analysis indicates that there are fewer than expected hub PPIs that involve fully disordered proteins. These are the dark blue colored regions on the far right and at the top of Figure 1.

To sum up, only a relatively small portion of proteins involved in the hub PPIs in human, including 17% of hubs and 11% of their interactors, are enriched in the intrinsic disorder. This enrichment is in line with an overall increase in the disorder content among the hubs that was also reported in prior studies [10,47,48,59,61]. The proteins that participate in the high-density disorder-enriched hub PPIs harbor substantial amounts of disorder (over 50% of their residues are disordered) but they include fewer than expected fully disordered proteins.

### 2.2. Functional and Structural Characteristics of Proteins Involved in the Disorder-Enriched Hub Protein-Protein Interactions

We focus our analysis on the hubs and their interactors that are involved in hub PPIs and which have higher than expected density in the two-dimensional intrinsic disorder space. This group of PPIs has a density that is higher than expected by at least 50% (regions colored from orange to red in Figure 1) and includes hubs and interactors that have high levels of intrinsic disorder. Furthermore, besides analyzing the complete set of all interactors, we also consider a subset of interactors that excludes hubs. In other words, in the latter case we exclude interactors involved in the hub-hub interactions. We analyze a comprehensive set of functional and structural characteristics to uncover which of them, if any, is specific to a given group of proteins (hubs, all interactors, and interactors that exclude hubs) that are involved in the disorder enriched hub PPIs. We accomplish that by comparing the quantity of a given characteristic between the proteins involved in these disorder-enriched hub PPIs and proteins involved in the remaining PPIs (Table 1).

**Figure 2:**
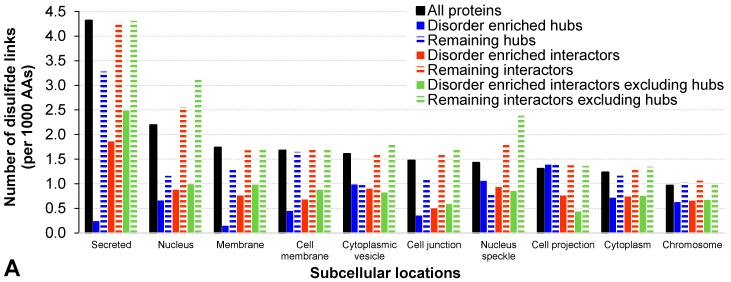

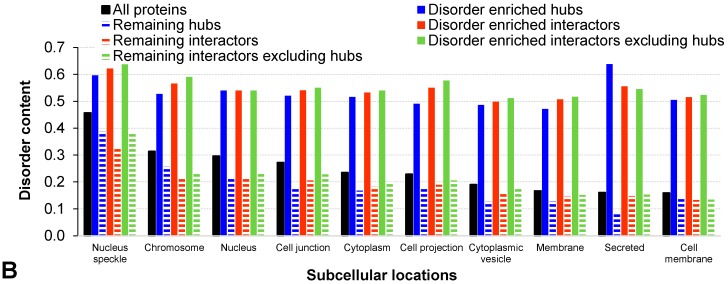
Number of disulfide bonds panel (**A**) and disorder content panel (**B**) in specific subcellular locations. We report the values for all human proteins (black bars), hubs (solid blue), hub interactors (solid red), and hub interactors that exclude hubs (solid green) that are associated with the disorder-enriched hub PPIs vs the remaining hubs (blue horizontal stripes), hub interactors (red horizontal stripes), and hub interactors that exclude hubs (green horizontal stripes), respectively, for each location. We consider all locations that include at least 10 proteins for each of the seven protein sets. The locations in panel (**A**,**B**) are sorted in descending order by the values of the number of disulfide bonds (disorder content) for all proteins (black bars).

As expected, both hubs and the two sets of hub interactors that are engaged in the high-density and disorder enriched hub PPIs are characterized by a significantly higher amount of intrinsic disorder (*p*-value < 0.001; see “Structural properties” rows in Table 1). The corresponding relative enrichment is >130% when quantifying the disorder by both content and normalized number of the long IDPRs. These observations are further supported by the significantly lower number of disulfide links, which can be used as a proxy for the formation of a stable tertiary structure, among the hubs and hub interactors that are engaged in the disorder-enriched hub PPIs (*p*-value < 0.001, decrease by over 46%). We break down this analysis based on the subcellular location of these proteins. We annotate the locations based on the Universal Protein resource (UniProt) tags using a protocol defined in [64]. We analyze the results for all considered human proteins, hubs, hub interactors, and hub interactors that exclude hubs; the latter three proteins sets are subdivided into proteins associated with the disorder-enriched hub PPIs versus the remaining proteins. To ensure statistically sound estimates of the disorder content and number of disulfide bonds, our analysis includes 10 cellular locations for which we found at least 10 proteins for all considered protein sets, see Figure 2. As expected, we observe that the counts of the disulfide bonds vary between the subcellular locations, with substantially larger numbers for secreted proteins when compared to the other cellular compartments (black bars in Figure 2A). This is in agreement with prior research, which similarly showed enrichment in the eukaryotic proteins associated with secretory pathways and lower abundance in the other locations, in particular in the cytoplasm [65]. More importantly, the disorder-enriched protein sets are characterized by the higher numbers of the disulfides (blue, red, and green bars with horizontal stripes in Figure 2A) compared to the corresponding sets of the proteins that exclude the disorder-enriched proteins (solid blue, red, and green bark in Figure 2A). Figure 2B confirms the expected trend that proteins involved in the disorder-enriched PPIs are characterized by the increased levels of disorder (solid bars are higher than the corresponding striped bars). It also show that the levels of the disorder content vary between subcellular compartment, with the highest values being found in the nucleus and lowest levels being among the membrane and secreted proteins. This is in agreement with the results shown in several recent studies [25,50,66].

Interestingly, Table 1 reveals that proteins associated with high-density disorder-enriched hub PPIs are characterized by similar evolutionary conservation levels and size when compared to the other proteins; the difference is below 20%. However, while the levels of conservation of the long disordered regions are similar for the hubs and the set of all their interactors, they are substantially lower, by over 70%, for the long IDPRs in interactors that exclude hubs and that are not part of the high-density disorder enriched hub PPIs. This finding is related to a recent observation that disordered regions in hubs are enriched in the conserved residues [67]. Furthermore, our results suggest that the long IDPRs in these disorder-depleted interactors are not only less evolutionarily conserved but also less likely to be involved in the PPIs; their content of the disordered protein binding (DPB) regions in Table 1 is much lower than that for the other protein sets.

The high-density disorder-enriched hubs and their interactors have significantly more MoRF (molecular recognition feature) and DPB (disordered protein binding) regions (*p*-value < 0.001; increase by over 50% and over 130%, respectively). This is likely due to the overall enrichment in the intrinsic disorder. As expected, the content of putative MoRFs is overall substantially lower than the content of the putative DPB regions. This is because MoRFs are a subset of the DPB regions. We emphasize that the abundance of the DPB regions in the interactors that exclude hubs is lower when compared to the hubs and the interactors that include hubs. More specifically, the DPB content equals 0.23 (the normalized number of protein binding regions equals 3.7) for the latter protein sets vs. 0.20 (3.3) for the interactors that exclude hubs. We also further investigate the relation between the MoRFs and the DPB regions given their close functional relation. The average point biserial correlation coefficients, which quantify relations for binary variables, that are measured between the predicted MoRF and DPB residues over the hub proteins, their interactors and the interactors that exclude hubs are equal to 0.24, 0.22, and 0.21, respectively. Furthermore, the Pearson correlation coefficients calculated between the per protein MoRF and DPB content value for the hubs, interactors, and interactors that exclude hubs are equal to 0.32, 0.33, and 0.33, respectively. Both sets of measurements suggest a weak correlation between the two related functional aspects of IDPRs. This expected observation to some extent validates the corresponding predictions collected from the two methods, MoRFpred and DisoRDPbind.

Furthermore, results based on the analysis of the abundance of a wide range of functional motifs defined with ELMs show that proteins linked with the high-density disorder enriched hub PPIs feature a modest decrease by 30% to 40% in the number of cleavage and degradation sites. More importantly, they also have significantly more catalytic sites (*p*-value < 0.001, increase by over 80%) and posttranslational modification (PTM) sites (*p*-value < 0.001, increase by over 110%). Furthermore, we observe divergent results concerning the non-catalytic binding sites and targeting sites between the interactors that exclude hubs and the other two sets of proteins, hubs and interactors that include hubs. While the abundance of the non-catalytic binding sites does not differ between the disorder-enriched and remaining proteins for the hubs and the set of all interactors, it drops significantly by over 170% for the disorder-depleted interactors that exclude hubs. The number of targeting sites for the interactors that exclude hubs is very low (near zero) when compared to a relatively high number of these sites for hubs. Furthermore, the number of the targeting sites is significantly increased in the disordered-enriched hubs/hub-including interactors compared to the remaining hubs/hub-including interactors (*p*-value < 0.001, increase by 150%).

PTMs extend the range of physico-chemical properties of amino acids, thereby diversifying structures and functions of target proteins, affecting their conformational stabilities, modulating their interactions with nucleic acids, membranes, ligands or other proteins, and targeting modified proteins to specific subcellular compartments [68,69,70]. In fact, the presence of various PTMs (as many as 300 different PTMs are known to occur physiologically [71]) extends the repertoire of amino acids found in proteins from 20 primary residues utilized during protein biosynthesis to more than 140 physico-chemically different residues, thereby dramatically extending the complexity of eukaryotic proteomes [68]. PTMs can affect both structured proteins/regions and IDPs/IDPRs. PTMs that preferentially affect IDPs/IDPRs are common in proteins engaged in regulatory and signaling functions. These disorder-associated PTMs were shown to include acetylation, acylation, adenylation, ADP ribosylation, amidation, carboxylation, formylation, glycosylation, methylation, sulfation, phosphorylation, prenylation, ubiquitination, and Ubl-conjugation [72]. Previous bioinformatics analyses revealed that protein phosphorylation sites are often found in IDPRs [73,74,75], and there is a high correspondence between the prediction of disorder and the occurrence of phosphorylation [75]. In addition to phosphorylation, several other types of PTMs, such as acetylation, protease digestion, ubiquitination, fatty acid acylation, and methylation, have also been observed to occur in IDPRs [72,73,74,76].

The analysis using the PTMs annotated with ModPred [77], which is independent from and extends the analysis based on ELMs, confirms the enrichment in the number of PTM sites and the number of residues that have PTM sites. We computed both quantities since some residues may have multiple PTM sites. Table 1 provides a detailed breakdown of this enrichment across the 21 major PTMs. The hubs and their interactors (including the set of interactors without hubs) that are involved in the high-density disorder-enriched hub PPIs have a significantly larger number of acetylation, ADP-ribosylation, amidation, carboxylation, hydroxylation, methylation, O-linked glycosylation, phosphorylation, sulfation, and ubiquitination sites. Our results specifically concerning proteins that participate in the disorder-enriched hub PPIs are in good agreement with the prior analyses of a more generic set of IDPRs [72,73,74,75,76]. Interestingly, MoRFs in the eukaryotic proteins were shown to be significantly enriched in PTM sites, suggesting that PTMs are crucial for the modulation of MoRF-driven protein recognition related to transcriptional and developmental processes [77]. This provides a feasible explanation for our observation that both MoRFs and certain PTM sites are significantly more abundant among our proteins of interest.

**Figure 3:**
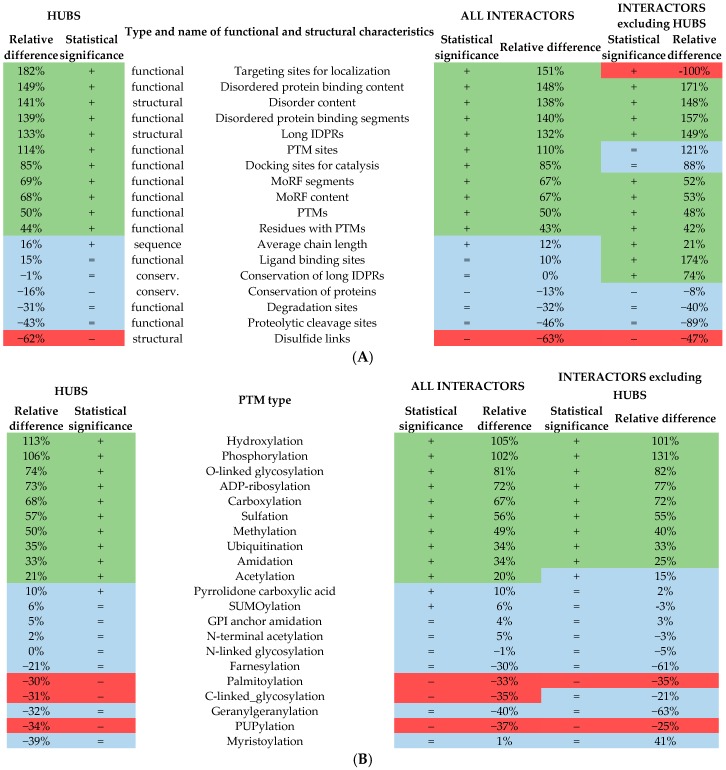
Significance of the differences in the functional and structural characteristics between hubs (on the left), all hub interactors, and hub interactors that exclude hubs (on the right) that are associated with the disorder-enriched hub PPIs when compared to the remaining hubs (on the left) and the corresponding remaining interactors (on the right). Panel (**A**) summarizes the results concerning structural characteristics, functional regions and motifs, evolutionary conservation and the overall abundance of PTMs. Panel (**B**) gives detailed results for specific types of PTMs. We reported relative differences and their statistical significance. The characteristics are sorted in descending order by their relative differences for the hubs. The characteristics are color-coded as follows: green for large (relative difference > 20%) and statistically significant (*p*-value < 0.001) enrichment; red for large (relative difference < −20%) and statistically significant (*p*-value < 0.001) depletion; and blue for lack of large and significant differences (|relative difference| < 20% or *p*-value over 0.001). Abbreviations: Eukaryotic linear motif (ELM); molecular recognition feature (MoRF; short disordered protein binding region); and posttranslational modification (PTM).

Figure 3 summarizes the results from Table 1 and reveals that they are similar between the hubs and their interactors that include hubs. It lists the considered functional and structural features that are sorted in descending order by the value of their relative difference between the proteins involved in the disorder-enriched hub PPIs and the other proteins. Figure 3A shows the characteristics that have the statistically significant and highest levels of enrichment among the disorder-enriched hubs and interactors that include hubs contain a substantial increase in the amount of DPB regions, a larger number of long IDPRs, and a significantly increased content of intrinsic disorder. These characteristics also include considerably increased rate of targeting, catalytic, and PTM sites. Figure 3B reveals that the hydroxylation, phosphorylation, O-linked glycosylation, ADP-ribosylation, carboxylation, sulfation, and methylation sites are the most enriched PTMs among the hubs and all their interactors that participate in the disorder-enriched hub PPIs. Furthermore, the far right part of Figure 3 reveals that the patterns of enrichment are different for the interactors that exclude hubs. They are characterized by low abundance of the targeting sites, which nears zero for the disorder-enriched interactors. These disorder-enriched interactors also have significantly more ligand binding sites and are more conserved when compared to the disorder-depleted interactors. However, the characteristics of the PTM sites are similar. 

### 2.3. Proteins Involved in the Disorder-Enriched Hub Protein-Protein Interactions and Human Diseases

We analyzed whether the abundance of hubs and their interactors that participate in the disorder-enriched hub PPIs is biased among a subset of human proteins that are associated with diseases. Table 2 summarizes these results. When considering the complete set of over 4000 disease-linked proteins, we show that they are significantly enriched among the proteins that participate in the high-density disorder-enriched hub PPIs (*p*-value < 0.001, 20% increase when compared to the human proteome). Analysis that focuses on the 18 major classes of diseases, each associating with at least 100 human proteins, reveals a significant enrichment for 11 disease classes, significant depletion for five classes and no significant differences for the remaining two classes. We observed the largest enrichment by over 600% in the disorder-enriched hub PPIs for the neoplasm-centered diseases that include a variety of cancers (MeSH class C04). Several other classes of diseases are also characterized by high values of over 50% enrichment. These include stomatognathic maladies (C07) and diseases of the endocrine system (C19), digestive system (C06), respiratory tract (C08), female urogenital system (C13), nervous system (C10), and musculoskeletal diseases (C05).

These observations are in line with the results of prior rigorous investigations of IDP/IDPR functions and dysfunctions that already have revealed that intrinsic disorder is prevalent among the disease-related proteins, with dysfunction of IDPs being often related to the development of various pathological conditions [30,31,32,33,35,36,78,79]. Many diseases are correlated with proteins predicted to be disordered, whereas no disease-associated proteins were found to be correlated with the absence of disorder [72,80,81]. In agreement with our results, the prevalence of IDPs/IDPRs was illustrated for various human diseases, such as cancer [82] (MeSH class C04), cystic fibrosis [83] (C06, C08 and C16) and neurodegenerative diseases [30] (C10). Among the cancer associated proteins with experimentally confirmed IDPRs are p53 [84], BRCA1 [85], EWS [86], HPV proteins [87], PTEN [88], and cancer/testis antigens (CTAs) [89]. The most notable disordered players associated with the pathogenesis of neurodegeneration are α-synuclein (Parkinson’s disease (PD), dementia with Lewy bodies, Alzheimer’s disease (AD), Down’s syndrome, and other synucleinopathies) [46], amyloid β and tau protein (AD), prions (Creutzfeldt-Jakob disease, scrapie, bovine spongiform encephalopathy), ataxin (Spinocerebellar ataxia) [30], as well as TAR-DNA binding protein-43 (TDP-43), RNA-binding protein FUS, polypeptides generated as a result of their intronic hexanucleotide expansions of the cofilin-binding protein C9orf72, and profilin-1 (PFN1) associated with amyotrophic lateral sclerosis (ALS) and frontotemporal lobar degeneration [90].

High prevalence of intrinsic disorder in proteins associated with the various human diseases is related (at least in part) to the functional roles of IDPs/IDPRs in regulation and control of biological processes. In fact, it was pointed out that the abundant involvement of IDPs/IDPRs in various signaling, regulation, and recognition processes is defined by a broad set of functional advantages of IDPs/IDPRs over their ordered counterparts [21,91,92]. These advantages include (but are not limited to) the ability of IDPs/IDPRs to react easily and quickly and change their conformation in response to changes in the environment, their structural plasticity and conformational adaptability, their binding promiscuity, and their unique capability to fold differently while interacting with different binding partners [60,93]. These same factors also define the capability of IDPs/IDPRs to play diverse roles in the modulation and control of functions of their binding partners and in promotion of the assembly of supra-molecular complexes. Furthermore, the ability of IDPs/IDPRs to return to the highly flexible conformations after the completion of a particular function and their predisposition to adopt different conformations depending on their environment are their unique physiological properties. They define the ability of IDPs/IDPRs to have different functions in different cellular contexts according to a specific conformational state [21]. Furthermore, in line with our observations in this study, biological activities of IDPs/IDPRs can be precisely and tightly controlled and regulated by various PTMs [21,77,94,95] and by alternative splicing (AS) [29,96,97]. In fact, the ability of AS to generate multiple protein isoforms with highly diverse sets of regulatory elements [96] is determined by the mosaic structure of IDPs/IDPRs that are known to contain multiple relatively short, functional elements, which, being spread within the amino acid sequences, define the multi-functionality of these proteins [98]. Clearly, AS-driven removal of pieces of an IDP/IDPR sequence containing different functional elements could dramatically reshuffle such multi-functionality [29,97,99].

The aforementioned factors define the capacity of IDPs/IDPRs to serve as hubs and as important controllers and regulators of various biological processes. However, all these factors also suggest that IDPs and hybrid proteins have to be tightly regulated and controlled themselves, since controllers/regulators have to be available in appropriate amounts and not to be present longer than needed [34,36,98]. In fact, there are numerous studies that not only emphasized the important roles of disordered regulators in signaling [100,101], regulation [28,102,103,104,105,106,107,108], cell protection [109], protein protection [110,111], and cellular homeostasis [112,113], but which also showed that IDPs/IDPRs are concisely controlled by themselves via multiple mechanisms, such as interaction with chaperones [114,115,116,117,118] or nanny proteins [119], partner binding [120,121,122,123,124,125,126], various PTMs [127,128,129,130,131], and regulated degradation [132,133,134,135,136,137]. Obviously, the consequences of a tightly controlled process, which suddenly goes out of control, could be disastrous. Similarly, the consequences of the misbehavior of an important regulator are disastrous too, and the development of particular pathological conditions is often linked to the failure of a specific peptide or protein (or a set proteins and peptides) to adopt its functional conformational state. These maladies are commonly known as conformational diseases, each originating from the dysfunction and misbehavior of a particular protein (or a set of proteins). Some disease-related proteins are characterized by the ability to spontaneously form pathologic conformation(s), whereas misbehavior of other proteins is determined by their mutations or (impaired) interactions with chaperones, intracellular or extracellular matrices, other proteins, nucleic acids, small molecules, other endogenous factors, or by the exposure to internal or external toxins. Furthermore, impaired PTMs, wrecked AS, an increased probability of degradation, impaired trafficking, loss of binding partners, or oxidative damage can also be pathogenic. Finally, one should keep in mind that all these factors can act independently, additively, or synergistically [33].

### 2.4. Several Illustrative Examples of Proteins Involved in the Disorder-Enriched Hub Protein-Protein Interactions

The next few sections provide description of several illustrative examples of proteins involved in the disorder-enriched hub PPIs. Supplementary Materials Tables S1 and S2 list the 100 most and the 100 least disordered-enriched human hubs, respectively, and include information about their putative disorder content, normalized number of DPB regions, and degree (promiscuity). These tables also provide a list of all their interactors, interactors involved in the disorder enriched hub PPIs, and the disorder content for each listed interactor. Supplementary Materials Table S1 shows that binding promiscuity *N* is not directly correlated with protein disorderedness. In fact, one of the most disordered proteins in this dataset, the intracellular hyaluronan-binding protein 4 (UniProt ID: Q5JVS0) with the disorder content of 0.88, is characterized by a relatively low number of interactors (*N* = 29), for a hub protein. On the other hand, the most promiscuous binder in this set (*N* = 415), BAG family molecular chaperone regulator 3 (UniProt ID: O95817), is characterized by the disorder content of 0.86. Supplementary Materials Table S2 reveals that the most ordered hubs have anywhere between 25 and 8548 interactors. Therefore, we describe 15 representative hubs that are grouped into the three following categories: (a) the most disordered human hubs; (b) highly disordered human hubs with the largest number of interactors; and (c) highly connected ordered hubs. We note that the latter set of hubs by definition is not involved in the disorder enriched hub PPIs. This is why the Supplementary Materials Table S2 does not include the associated list of the interactors that participate in the disorder enriched hub PPIs.

**Figure 4:**
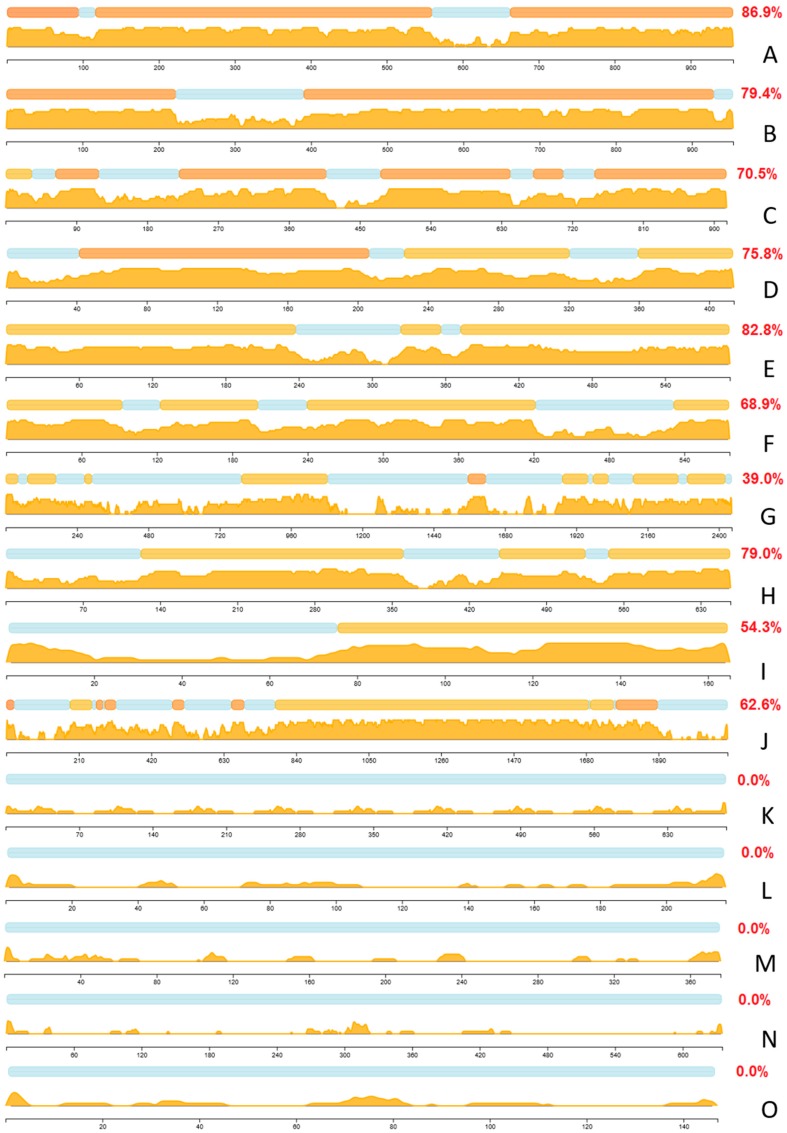
Intrinsic disorder levels in the disordered human hubs characterized by the highest levels of disorder (**A**–**E**), highly disordered hubs characterized by the highest levels of interactability (**F**–**J**), and ordered hubs with the highest interactability levels (**K**–**O**). The disorder was annotated using the MobiDB platform [141,142,143]; disorder content is shown in red font. Each plot represents disorder tendencies in two forms—by bar plots showing location of IDPRs and by area plots showing sequence distribution of consensus disorder scores evaluated by MobiDB lite disorder predictor [143]. (**A**) Thyroid hormone receptor-associated protein 3 (UniProt ID: Q9Y2W1). (**B**) Zinc finger CCCH domain-containing protein 18 (UniProt ID: Q86VM9). (**C**) Scaffold attachment factor B1 (UniProt ID: Q15424). (**D**) Intracellular hyaluronan-binding protein 4 (UniProt ID: Q5JVS0). (**E**) TATA-binding protein-associated factor 2N (UniProt ID: Q92804). (**F**) BAG family molecular chaperone regulator 3 (UniProt ID: O95817). (**G**) CREB-binding protein (UniProt ID: Q92793). (**H**) RNA-binding protein EWS (UniProt ID: Q01844). (**I**) Cyclin-dependent kinase inhibitor 1 (UniProt ID: P38936). (**J**) Mediator of DNA damage checkpoint protein 1 (UniProt ID: Q14676). (**K**) Ubiquitin (UniProt ID: P0CG48; 8548 interactors). (**L**) Growth factor receptor-bound protein 2 (UniProt ID: P62993; 804 interactors). (**M**) Actin (UniProt ID: P60709; 263 interactors). (**N**) Protection of telomeres protein 1 (UniProt ID: Q9NUX5; 200 interactors). (**O**) Protein mago nashi homolog (UniProt ID: P61326; 190 interactors).

Furthermore, we analyzed the putative DPB regions and disorder content for these proteins using not only the same tools that were used to characterize the entire human interactome, but also a second set of independent tools. Besides using DisoRDPbind [138,139], we also utilized ANCHOR [140] to predict putative disordered protein binding regions; we call these ANCHOR-identified binding sites (AIBSs). We annotated the putative disorder and computed disorder content using the MobiDB platform that applies eight methods to derive a consensus-based prediction [141,142,143], on the top of the analysis that is based on the consensus of five versions of IUPred [144] and ESpritz [145] methods that we utilized to annotate the human PPI network. The results of the disorder analysis with MobiDB for the selected 15 proteins are summarized in Figure 4. These proteins include five of the most disordered human hubs (Figure 4A–E), five highly disordered human hubs with the highest promiscuity (Figure 4F–J), and five highly connected ordered hubs (Figure 4K–O). The results with MobiDB are in good agreement with the results based on the other two predictors. The average disorder content equals 0.88 (for IUPred and Espritz-based analysis) vs. 0.79 (for MobiDB-based analysis) for the five most disordered hubs, 0.74 vs. 0.61 for the most promiscuous disordered hubs and 0 vs. 0 for the highly connected ordered hubs.

We also note the average disorder content values of the interactors of the selected hubs, which were computed with the consensus of five versions of IUPred and Espritz (Supplementary Materials Tables S1 and S2). These values range between 0.23 and 0.38 for the most disordered hubs, 0.19 and 0.34 for the most promiscuous hubs, and between 0.20 and 0.33 for the highly connected ordered hubs. As expected, the average disorder content of the interactors that participate in the disorder enriched hub PPIs is much higher and varies from 0.84 to 0.88 for the most disordered hubs and from 0.50 to 0.89 for the most promiscuous hubs.

#### 2.4.1. The Most Disordered Hubs

Curiously, the most disordered human hubs considered in this section are nuclear proteins. This observation is in line with the known fact that the nuclei of eukaryotic cells are often enriched in IDPs or hybrid proteins with long functional IDPRs [50,66]. Binding promiscuity of these mostly disordered nuclear hubs is driven by the presence of multiple long disorder-based protein binding regions. Supplementary Materials Table S1 shows that the 31 hubs that have disorder content >0.8 on average have 13 such regions per unit of 1000 residues while the average for the complete set of 100 most disordered-enriched human hubs equals 11. The normalized (per 1000 residues) number of these putative long DPB regions for the top 100 hubs is modestly correlated with the disorder content (Pearson correlation coefficient = 0.26). The subsequently discussed five proteins with the highest disorder content are sorted in descending order by their disorder content.

Thyroid hormone receptor-associated protein 3 (Supplementary Materials Table S1: UniProt ID Q9Y2W1; disorder content = 0.89; 72 interactors; average disorder content of all (disorder enriched) interactors = 0.29 (0.85)). RNA processing factor THRAP3 is a 955 residue-long arginine/serine-rich domain-containing nuclear protein involved in pre-mRNA splicing [146]. As many other highly disordered proteins, THRAP3 (which is also known as Trap150) has several functions ranging from regulation of signal-induced alternative splicing [147], nuclear mRNA decay [146], response to DNA damage [148], transcriptional co-activation [149], adipocyte differentiation [150], to the regulation of the circadian clock [151]. Together with another arginine/serine-rich domain-containing protein BCLAF1 (Bcl-2-associated transcription factor 1), as well as Hakai, Virilizer homolog, KIAA0853 (also known as zinc finger CCCH domain-containing protein 13, ZC3H13), RBM15 (RNA-binding motif protein 15), several general splicing regulators, and Wilms’ tumor 1-associating protein (WTAP), THRAP3 was shown to form the WTAP complex that serves as an important component of the RNA processing machinery [152]. Furthermore, THRAP3 seems to control WTAP localization to nuclear speckles [152]. Recently, the important role of the aberrant THRAP3 phosphorylation in androgen-independent prostate cancer cell growth was demonstrated including the presence of a remarkable difference in the interactability of the non-phosphorylated and phosphorylated forms of this protein [153]. In addition to phosphorylation, THRAP3 is known to undergo extensive and variable PTMs at numerous sites. N-terminal third of this protein shows strong compositional biases, being serine-arginine enriched, and therefore is known as arginine/serine-rich domain. According to the ANCHOR-based analysis, THRAP3 has 25 disorder-based protein-protein binding sites (AIBSs) that range in length from 6 to 58 residues (residues 1–56, 65–122, 162–180, 188–196, 220–265, 276–317, 445–351, 374–394, 408–426, 450–460, 470–479, 513–530, 549–569, 586–597, 609–614, 626–659, 675–694, 704–725, 784–793, 809–822, 830–844, 876–883, 903–919, and 921–928) and that cover 56.3% of its sequence. Therefore, with more than half of its sequence serving as a set of potential protein-protein binding sites and with high arginine and lysine content (11.9% and 10.5%, respectively), it is not surprising that THRAP3 serves as a highly connected hub protein that is also able to efficiently interact with RNA. Figure 4A represents a disorder profile generated for THRAP3 with MobiDB database. It is clear that THRAP3 is a highly disordered protein (disorder content = 0.869), which is in agreement with the disorder computed with IUPred and Espritz (disorder content = 0.892).

Zinc finger CCCH domain-containing protein 18 (Supplementary Materials Table S1: UniProt ID Q86VM9; disorder content = 0.89; 40 interactors; average disorder content of all (disorder enriched) interactors = 0.31 (0.88))*.* ZC3H18 is a 953 residue-long metal binding protein containing a C_3_H_1_-type zinc finger motif (residues 219–245) and interacting with RNA, likely due to its high arginine (11.6%) and lysine (8.2%) content. This protein contains three coiled coil regions (residues 105–134, 399–464, and 921–950), a poly-asparagine region (residues 201–204), and two regions of high compositional bias (proline- and serine-regions at residues 259–296 and 532–746, respectively). Similar to THRAP3, ZC3H18 protein is enriched in disorder-based protein-protein interaction sites. It contains 22 AIBSs that range in length from 8 to 78 residues, are uniformly spread over the ZC3H18 sequence, and which account for 60.8% of its amino acids (residues 1–11, 15–60, 78–102, 108–154, 167–183, 195–203, 222–258, 273–287, 304–315, 365–393, 502–516, 522–553, 561–569, 574–604, 616–623, 630–641, 648–656, 680–751, 781–793, 803–838, 856–871, and 876–953). There are multiple different PTMs such as phosphorylation, acetylation and SUMOylation in ZC3H18, suggesting that interactivity of this highly disordered promiscuous hub (see Figure 4B) is PTM-controlled. In fact, ZC3H18 was shown to interact with the hepatitis B virus post-transcriptional element (PRE), which is one of the viral RNA elements facilitating mRNA export, and with proteins of the conserved TREX complex [154], which is engaged in coupling of the transcription with messenger RNA export [155].

Scaffold attachment factor B1 (Supplementary Materials Table S1: UniProt ID Q15424; disorder content = 0.88; 65 interactors; average disorder content of all (disorder enriched) interactors = 0.31 (0.87)). SAFB1 is a 915 residue-long DNA binding protein that is known to interact with the S/MAR DNA (scaffold/matrix attachment region). This interaction is responsible for coupling of transcription and RNA processing via the formation of a molecular assembly point needed for the formation of a transcriptosomal complex that includes RNA polymerase II and SR proteins involved in the RNA splicing and containing domains with long serine-arginine repeats [156]. SAFB1 is a member of a protein family that includes SAFB2 and the SAFB-like transcriptional modulator SLTM [156]. Human SAFB1 is predicted with MobiDB to be highly disordered (see Figure 4C) and has 18 AIBSs, some as long as 95 residues (residues 1–6, 12–21, 31–43, 54–65, 116–210, 212–228, 235–297, 306–341, 353–367, 404–414, 422–438, 446–455, 463–482, 560–582, 598–609, 728–747, 797–813, and 832–915). These disordered protein-protein binding regions cover 68.2% of SAFB1 sequence and are responsible for the ability of this protein to interact with multiple partners. In fact, C-terminally located domain (residues 528–792) is known to interact with the subunit A of RNA polymerase II (POLR2A) [157], whereas interaction with SAFB2 relies on an overlapping region spanning residues 599–915. Curiously, both of these regions contain multiple AIBSs. On the other hand, the N-terminal half of SAFB1 contains a SAP motif (residues 31–65) that includes two AIBSs and, in other nuclear proteins, is involved in transcription, DNA repair, RNA processing and apoptotic chromatin degradation. The presence of the two AIBSs in SAP motif is in line with the observation that this motif might form two alpha helices separated by a turn [158]. Also, SAFB1 contains a RNA recognition motif (RRM) located at the middle of the protein (residues 408–484) and possessing several AIBSs. Finally, there are three regions with strong composition biases, residues 612–831, 625–705, and 785–903, which are enriched in arginines, glutamates, and glycines, respectively. In addition to the canonical form, human SAFB1 might have three isoforms produced by alternative splicing and has multiple PTM sites.

Intracellular hyaluronan-binding protein 4 (Supplementary Materials Table S1: UniProt ID Q5JVS0; disorder content = 0.88; 29 interactors; average disorder content of all (disorder enriched) interactors = 0.38 (0.84)). IHABP4 (also known as Ki-1/57 intracellular antigen) is a 413 residue-long highly disordered (according to the analysis with MobiDB, see Figure 4) cytoplasmic and nuclear protein that plays a role in several nuclear functions, such as the chromatin remodeling and the transcription regulation [159,160]. The role of IHABP4 in regulation of transcription is supported by the ability of this protein to interact with the adaptor protein RACK1, the transcription factor MEF2C, and the chromatin remodeling factor CHD3 [160]. Furthermore, IHABP4 is engaged in interaction with eleven p53-binding proteins and can interact with p53 itself [159]. For its functional regulation, IHABP4 can be phosphorylated on several serine and threonine residues [160]. It has a coiled-coil domain (residues 40–64) and is predicted to have 11 AIBSs (residues 1–45, 56–88, 94–119, 133–169, 201–246, 254–262, 276–299, 317–336, 338–365, 384–400, and 408–413) that range from 6 to 46 residues in length and cover 70.5% of the IHABP4 sequence. It seems that all these AIBSs can be engaged in interaction with various binding partners. In fact, N-terminal region (residues 1–150) where shown to bind to UBC9, GADD34, YB-1, and SF2p32, whereas the C-terminal region (residues 122–413) is responsible for interaction with RACK1, CHD-3, p53-binding protein, LUN, DAXX, PIAS-3, HNG-protein 2-like 1, and Tip-60 [159]. On the other hand, the entire IHABP4 sequence is needed for interaction of this protein with p53 [159].

TATA-binding protein-associated factor 2N (Supplementary Materials Table S1: UniProt ID Q92804; disorder content = 0.88; 54 interactors; average disorder content of all (disorder enriched) interactors = 0.24 (0.87)). TAF15 (formerly known as TAF_II_68) is a 592 residue-long nuclear RNA-binding protein that can also interact with single stranded DNA (ssDNA). Functionally, TAF15 contains an N-terminal activation domain, an RNA recognition motif (RRM, residues 234–320), and numerous (at least 21) Arg-Gly-Gly (RGG) repeats that spread throughout its C-terminal end (residues 407–575) [161]. These RGG repeats are needed for RNA binding of this protein [162]. TAF15 is structurally related to TLS/FUS and EWS proteins, and together these multifunctional proteins constitute the TET family [163]. Each of the TET family members is able to contribute a potent transcriptional activation domain to oncogenic fusion proteins originating due to the chromosomal translocation and often serving as the primary causes of their associated cancers [163]. In fact, in certain human chondrosarcomas, the N-terminal activation domain of TAF15 serves as an essential transforming domain in the fusion oncoproteins created by chromosomal translocation [164]. This N-terminal transactivation domain of TAF15 was shown to possess transactivation and oncogenic properties [165]. Figure 4E shows that TAF15 is another highly disordered protein according to the MobiDB-based analysis. There are eight AIBSs in TAF15, which are exclusively located within the N-terminal transactivation domain (residues 16–22, 28–84, 98–192, 204–218, 234–261, 297–321, 358–373, and 394–401). Therefore, although AIBSs cover 42.3% of the full-length TAF15, these disorder-based protein-protein interaction sites occupy 62.6% of its 401 residue-long activation domain.

#### 2.4.2. Highly Connected Hubs Enriched in Disorder

The section below analyses the most connected human hubs that contain high levels of intrinsic disorder. Disordered hubs in this section are sorted by their interactability (number of interactors).

BAG family molecular chaperone regulator 3 (Supplementary Materials Table S1: UniProt ID O95817; 415 interactors; disorder content = 0.86; average disorder content of all (disorder enriched) interactors = 0.19 (0.85)). High binding promiscuity of this highly disordered BAG3 protein (see Figure 4F for the MobiDB-based result) can be defined by the fact that this 575 residue-long protein serves as a co-chaperone for HSP70 and HSC70 chaperone proteins [166]. BAG3 is a nucleotide-exchange factor (NEF), the major function of which is the promotion of the release of ADP from the HSP70 and HSC70 proteins required for triggering the release of client/substrate proteins [167]. BAG3 acts as a two-sided sword, promoting nucleotide release via binding to the nucleotide-binding domain (NBD) of HSPA8/HSC70, and mediating the substrate release via interaction with the substrate-binding domain (SBD) of HSPA8/HSC70 [166,167]. Human BAG3 contains two WW domains (residues 20–54 and 124–154, also known as rsp5 or WWP domains), which are shown to be engaged in interaction with proteins containing specific proline-motifs, [AP]-P-P-[AP]-Y and phosphorylated serine/threonine-proline sites, often mediating the assembly of multiprotein networks [168]. Among other specific features of the human BAG3 amino acid sequence are the presence of a short poly-serine region (residues 180–187) and another functional domain, BAG domain (residues 421–498), needed for interaction with HSP70 and HSC70 and positive or negative regulation of these chaperone proteins [166]. According to the ANCHOR analysis, 73.6% of BAG3 sequence can be engaged in disordered protein-protein interactions. There are twelve AIBSs in human BAG3 (residues 1–17, 22–46, 81–141, 147–164, 173–314, 329–350, 362–371, 383–401, 420–439, 450–466, 490–537, and 552–575). Importantly, the known functional domains of BAG3 either overlap with AIBSs (e.g., WW-1 and WW-2) or include such disorder-based binding sites (e.g., BAG domain that contains AIBSs spanning residues 420–439 and 450–466).

CREB-binding protein (Supplementary Materials Table S1: UniProt ID Q92793; 262 interactors; disorder content = 0.63; average disorder content of all (disorder enriched) interactors = 0.34 (0.51)). CREBBP (or CBP) is a 2442 residue-long protein found both in the nucleus and cytoplasm with a wide spectrum of important biological functions. These functions range from histone acetyltransferase (HAT) activity [169], to acetylation of proliferating cell nuclear antigen (PCNA) [170] and many other non-histone proteins, such as general transcription factors [171], erythroid Krueppel-like factor (EKLF) [172], forkhead transcription factor FOXO1 [173] and many other proteins, to regulation of the cell cycle [174], to promiscuous interaction with multiple protein partners, including CREB [175], as well as hypoxia-inducible factor 1α (HIF-1α), NF-κB, p53, and STAT proteins [176], among many others. Because of this multifunctionality and binding promiscuity, CREBBP and its paralogue p300 are considered as central nodes in eukaryotic transcriptional regulatory networks [177]. Human CREBBP is a large multidomain protein containing several ordered domains (TAZ1 (residues 352–432), KIX (residues 587–666), bromodomain BRD (residues 1094–1180), CH2 (residues 1192–1318), HAT (residues 1323–1700), ZZ (residues 1701–1742), TAZ2 (residues 1770–1843), and nuclear coactivator binding domain (NCBD, residues 2059–2115) accounting for ~950 of its residues, with the remaining ~1500 residues being intrinsically disordered [176] (see Figure 4G). The importance of intrinsic disorder for functions and interactions of CREBBP was systemized in a recent review [176]. The ability of CREBBP to be engaged in numerous protein-protein interactions is defined by the presence of 34 AIBSs (residues 1–7, 17–102, 109–140, 145–165, 171–221, 232–247, 254–283, 295–314, 353–365, 479–500, 515–578, 599–613, 628–635, 649–662, 673–683, 707–768, 776–879, 881–898, 902–1004, 1017–1059, 1084–1102, 1121–1127, 1537–1551, 1616–1639, 1844–1851, 1857–1878, 1911–1943, 1952–2028, 2032–2054, 2060–2077, 2084–2114, 2132–2200, 2217–1353, and 2371–2442) that involve 1304 (or 53.4%) residues. AIBSs are abundantly present in disordered linker regions connecting globular domains of this important protein. In fact, it seems that almost all residues in linker regions can be used for the protein-protein interactions. Furthermore, it was emphasized that even ordered domains of CREBBP show high levels of interactability serving as scaffolds that bind disordered transactivation domains of a wide variety of partners [176].

RNA-binding protein EWS (Supplementary Materials Table S1: UniProt ID Q01844; 227 interactors; disorder content = 0.81; average disorder content of all (disorder enriched) interactors = 0.27 (0.84)). EWS oncogene (also known as Ewing sarcoma breakpoint region 1 protein) is a 656 residue-long nuclear and cytoplasmic protein that can also be found in cell membrane [178]. Similar to TAF15, EWS is highly disordered (see Figure 4H; disorder content = 0.79 according to the MobiDB analysis) member of the TET protein family [163]. N-terminal half of this protein contains 31 approximate tandem repeats and is considered as EAD (Gln/Pro/Thr-rich) region. EWS also has a short IQ domain (residues 256–286), an RRM domain (residues 361–447), and three C-terminally located Arg/Gly/Pro-rich regions (residues 300–340, 454–513, and 559–640). Also, similar to TAF15, EWS often serves as an important part of the fusion oncoproteins created by chromosomal translocation [179,180,181]. High disorder levels in EWS and TAF15 are in line with the important notion that many oncogenic fusion proteins are enriched in intrinsic disorder, and that this high disorderedness enables these proteins to evade cellular surveillance mechanisms evolved for the elimination of misfolded proteins [182]. According to the ANCHOR analysis, human EWS contains 14 AIBSs (residues 89–98, 110–131, 156–185, 200–234, 242–293, 300–336, 343–350, 360–388, 396–403, 423–460, 465–473, 482–501, 512–559, and 570–644) that cover 64.2% of its sequence. Since almost the entire sequence of EWS (except to the 88 N-terminal residues) is densely packed with these disorder-based binding sites, it is likely that they do play important roles in the promiscuity of this protein.

Cyclin-dependent kinase inhibitor 1 (Supplementary Materials Table S1: UniProt ID P38936; 206 interactors; disorder content = 0.63; average disorder content of all (disorder enriched) interactors = 0.20 (0.50)). CDK-interacting protein 1 or p21^Waf1/Cip1/Sdi1^ is a highly disordered (see Figure 4I; disorder content = 0.54 according to the MobiDB analysis) [183], 164 residue-long protein mediating G1/S-phase arrest through inhibition of cyclin-dependent kinases (Cdks), transcriptional regulation, modulation or inhibition of apoptosis, and playing an important role in controlling the p53-dependent cell growth and regulating the p53-mediated inhibition of cellular proliferation in response to DNA damage [102,105,106,184,185]. The prevalence of intrinsic disorder in p21 and another cyclin-dependent kinase (Cdk) regulator, p27, is well documented, and the importance of intrinsic disorder for functionality of these two proteins was systematically analyzed; see [102,105,106] for the topical reviews. Human p21 contains five AIBSs (residues 36–41, 68–79, 100–105, 109–123, and 145–164) accounting for 36.0% of its amino acid sequence. The very C-terminal AIBS coincides with the PIP-box K+4 motif that is known to mediate the p21 interaction with PCNA [186].

Mediator of DNA damage checkpoint protein 1 (Supplementary Materials Table S1: UniProt ID Q14676; 176 interactors; disorder content = 0.74; average disorder content of all (disorder enriched) interactors = 0.24 (0.89)). MDC1 (also known as nuclear factor with BRCT domains protein 1, NFBD1 or KIAA0170) is a 2089 residue-long, highly disordered (see Figure 4J; disorder content = 0.63 according to the MobiDB analysis) nuclear protein that serves as a key regulator of the DNA damage response [187]. As it follows from its name, MDC1 plays a crucial role in controlling the checkpoint mediated cell cycle arrest in response to DNA damage within both the S phase and G2/M phases of the cell cycle [188,189,190,191,192,193,194]. MDC1 contains an N-terminal FHA (forkhead-associated) domain (residues 54–105) and a tandem repeat of BRCT (breast cancer susceptibility gene-1 C terminus) domains (residues 1892–1970 and 1991–2082). The tandemly repeated BRCT domains of MDC1 are required for localization of this protein to chromatin [193]. There are several functional regions in this protein needed for MDC1 interaction with the CHK2 (residues 1–150), the MRN complex (residues 1–220), or the PRKDC complex (residues 1148–1610). Furthermore, there are two nuclear localization signals (NLS1 and NLS2, residues 145–568, and NLS2, residues 1698–2089) and a proline-rich region (residues 1034–1469). Among all illustrative proteins considered in this section, human MDC1 is predicted to contain the largest number of AIBSs (43) that cover 1270 of its residues (or 60.8%). In fact, according to ANCHOR analysis, the DPB regions densely populate almost entire MDC1 sequence, with the noticeable exception for its N-terminal region (residues 1–200) that is predicted to be mostly ordered and to have only two short AIBSs (residues 1–8 and 32–37). On the other hand, the remaining 90% of this protein have AIBSs that range in length from 6 to 81 residues and are characterized by the mean length of 30 residues (residues 202–220, 235–265, 276–293, 314–332, 338–375, 384–403, 409–431, 434–445, 463–485, 503–522, 531–542, 556–577, 587–595, 604–618, 627–656, 686–760, 767–786, 796–824, 834–846, 862–868, 889–897, 907–946, 968–1045, 1051–1126, 1132–1174, 1182–1216, 1226–1255, 1260–1297, 1311–1341, 1352–1377, 1389–1421, 1430–1461, 1472–1507, 1518–1542, 1557–1615, 1628–1708, 1713–1747, 1754–1821, 1831–1841, 1893–1902, and 1909–1914).

#### 2.4.3. Highly Connected Hubs Depleted in Disorder

The proteins considered below are very different from the highly disordered human hubs presented in two preceding sections. They are examples of highly connected ordered hubs with no putative disorder; the disorder content is predicted to be 0. At first glance, the presence of such highly connected but ordered hubs may seem a contradiction to the major moto of this article “disorder is crucial for binding promiscuity”. However, one of the first studies dedicated to the analysis of roles of intrinsic disorder in PPI networks pointed out that intrinsic disorder can contribute to protein binding promiscuity in three general ways: “First, intrinsic disorder can serve as the structural basis for hub protein promiscuity; secondly, intrinsically disordered proteins can bind to structured hub proteins; and thirdly, intrinsic disorder can provide flexible linkers between functional domains with the linkers enabling mechanisms that facilitate binding diversity” [61]. We emphasize the second mechanism that provides an explanation for how structured hubs can be highly promiscuous by interacting with IDPs and IDPRs. To this end, Supplementary Materials Table S2 reveals that interactors of the highly connected structured hubs have substantial levels of disorder. The average computed over the average disorder content of interactors of the top 100 structured hubs equals 0.19. Moreover, the average disorder content for the interactors of the five protein ranges between 0.20 and 0.33 is described below.

Ubiquitin (Supplementary Materials Table S2: UniProt ID P0CG48; 8548 interactors; average disorder content of interactors = 0.20). Among proteins considered in this article, ubiquitin is characterized by the largest PPI network. This is not too surprising, since protein ubiquitination is one of the more common PTMs in eukaryotic cells, and, as a matter of fact, is present in bacteria and other prokaryotes, where target proteins are modified with ubiquitin-like proteins, such as recently discovered ubiquitin Bacterial (UBact) [195] and prokaryotic ubiquitin-like protein (Pup) [196], respectively. The biological importance of ubiquitin is reflected in an intriguing fact that this protein is encoded by four different genes, *UBA52*, *RPS27A*, *UBB*, and *UBC*. A single copy of ubiquitin fused to the ribosomal proteins L40 and S27a is encoded by *UBA52* and *RPS27A* genes, respectively, whereas *UBB* and *UBC* genes code for polyubiquitin precursors with exact head to tail repeats of three and nine identical ubiquitin sequences. Also, ubiquitin is one of the more abundant eukaryotic proteins.

It is known that mature ubiquitin (which is a 76 residue-long globular protein) can exist in unbound form, or be covalently bound to target proteins, which can be mono- and polyubiquitinated. Ubiquitination is intensively used in several biological processes, such as directing proteasomal protein degradation [197,198], regulation of budding of retroviral virions [199], modulation of the activity of transcription factors [200], control of the receptor endocytosis and lysosomal trafficking [201], mediation of the Arc-dependent synaptic plasticity [202], regulation of the repair of DNA double strand breaks (DSBs) by the ubiquitin-dependent molecular unfoldase/segregase p97 [203], regulation of various signaling pathways, such as insulin [204] and TGF-β signaling [205], as well as initiating immune response [206], and many others functions. In fact, it is close to impossible to provide an exhaustive list of biological activities regulated by ubiquitination, since as of October 24, 2017 there were 19,541 related entries in PubMed.

**Figure 5:**
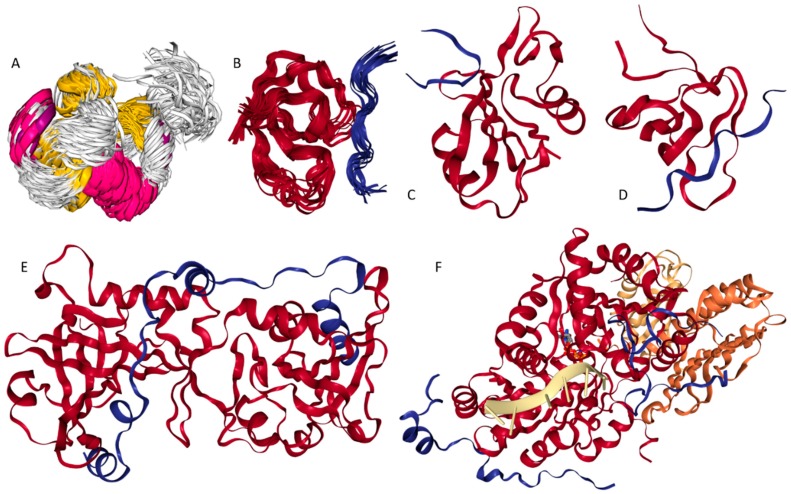
Structural characterization of highly connected ordered hubs. (**A**) Solution NMR structure of human ubiquitin (PDB ID: 1XQQ) [207]. (**B**) Solution NMR structure of a complex between the N-terminal SH3 domain of GRB2 (residues 1–56, red ribbons) and a peptide from SOS (blue ribbons) (PDB ID: 1AZE) [210]. (**C**) Minimized mean solution NMR structure of a complex between the SH2 domain of human GRB2 (residues 49–168, red ribbon) and a KPFY*VNVEF peptide (blue ribbon) (PDB ID: 1BMB) [211]. (**D**) Minimized mean solution NMR structure of a complex between the C-terminal SH3 domain of human GRB2 (residues 159–215, red ribbon) and a ligand peptide (blue ribbon) (PDB ID: 1IO6). (**E**) Crystal structure of a telomeric shelterin complex between the POT1 C-terminal domain (POT1C, residues 330–634, red ribbon) and POT1-binding region (residues 254–336, blue ribbon) of the adrenocortical dysplasia protein homolog (PDB ID: 5JUN7) [212]. (**F**) Crystal structure of a core EJC complex containing the complex of MAGOH (full length, dark orange ribbon), Y14 (residues 66–174, light orange ribbon), eIF4AIII (full length, red ribbon), Btz (the SELOR domain, residues 137–286, two blue ribbons), a non-hydrolyzable ATP analog (AMPPNP, bound to eIF4AIII), and U_15_ RNA (yellow ribbon) (PDB ID: 2J0Q) [213].

Although human ubiquitin is predicted to contain 0% of disordered residues (see Figure 4K), solution NMR analysis of this protein revealed that human ubiquitin is characterized by considerable conformational heterogeneity and can be described as a rather dynamic conformational ensemble (see Figure 5A) [207]. It was also pointed out that “the interior atoms of the protein are tightly packed in each individual conformation that contributes to the ensemble but their overall behaviour can be described as having a significant degree of liquid-like character” [207]. Curiously, although human ubiquitin has a unique structure with considerable conformational flexibility and heterogeneity, its prokaryotic homologue, Pup, is intrinsically disordered [208,209]. Finally, in relation to the subject of this study, it was indicated that the ubiquitination sites of target proteins are commonly located within IDPRs [76].

Growth factor receptor-bound protein 2 (Supplementary Materials Table S2: UniProt ID P62993; 804 interactors; average disorder content of interactors = 0.24). SH2/SH3 adapter protein GRB2 is a 217 residue-long regulatory protein found in nucleus, Golgi apparatus, endosomes, and cytoplasm [214]. The ability of GRB2 to affect various biological processes and modulate multiple proteins defines its position as a critical link between the cell surface growth factor receptors and downstream cellular targets [215,216]. Furthermore, GRB2 is able to inhibit receptor tyrosine kinases (RTKs, such as hepatocyte growth factor receptor and platelet derived growth factor receptor) that have important biological functions serving as basic communication systems between the extracellular milieu and intracellular signaling pathways [217], as well as it affecting non-receptor tyrosine kinases [218]. GRB2 conducts its regulatory activities by binding to specific phosphotyrosine-containing and proline-rich sequence motifs of countless target proteins. Among numerous binding partners of GRB2 are tyrosine phosphorylated son of sevenless (SOS) [219,220], tyrosine phosphorylated EGF and PDGF receptors [221,222], tyrosine phosphorylated SIT1, IRS1, IRS4, SHC, and LNK [223,224,225,226], phosphorylated C-terminus of SH2B2 [227], as well as phosphorylated LAX1, LAT, LAT2, and LIME1 [228,229,230,231]. GRB2 can also bind to NISCH, PTPNS1, REPS2 [232,233,234], and PIK3C2B [235].

Despite its remarkable binding promiscuity human GRB2 is characterized by a rather simple architecture, containing three functional domains, N-terminal SH3 domain (residues 1–58), SH2 domain (residues 60–152), and C-terminal SH3 domain (residues 156–217). Figure 5B–D shows NMR solution structures of these domains complexed with the corresponding target peptides. It is clearly seen that peptides bound to the functional domains of GRB2 are characterized by extended configuration, suggesting that they are mostly disordered in their unbound forms. This is in agreement with the known fact that SH3 and SH2 domains of human GRB2 prefer to bind to phosphotyrosine-containing and proline-rich sequence motifs, which are expected to be highly disordered. Therefore, ordered domains of GRB2 serve as an example of disorder-based many-to-one binding modes, where numerous disordered partners interact with the same ordered protein/domain.

Actin (Supplementary Materials Table S2: UniProt ID P60709; 263 interactors; average disorder content of interactors = 0.20). Actin, a 375 residue-long globular protein, is one of the most highly abundant proteins in every living cell. For example, in the muscle cells, actin concentration ranges from 230 to 960 μM [236]. Actin is known to possess unusual structural characteristics. Although its unique 3D-structure is stabilized by binding of one ATP molecule and one Ca^2+^ ion (or Mg^2+^ In Vivo), it is thermodynamically unstable and quasi-stationary [237,238], being formed in vivo via complex posttranslational folding processes that are assisted by Hsp 70, prefoldin, and chaperonin CCT and require the ATP energy expenditure. Among important functional features of actin is its ability to polymerize (to undergo G-F transition) and to interact with the other main muscle proteins, such as myosin, as well as with the regulatory proteins controlling muscle relaxation and contraction [239,240]. Furthermore, monomeric actin and actin-containing microfilaments are critically involved in numerous interactions with a wide spectrum of unrelated actin-binding proteins (ABPs) [241,242], many of which are characterized by high levels of intrinsic disorder [241].

Protection of telomeres protein 1 (Supplementary Materials Table S2: UniProt ID Q9NUX5; 200 interactors; average disorder content of interactors = 0.27). POT1 is a 634 residue-long nuclear protein that serves as an important constituent of the telomerase ribonucleoprotein (RNP) complex, also known as the shelterin complex (telosome), essential for the regulation and maintenance of telomere length and containing TERF1, TERF2, TINF2, TERF2IP, ACD, and POT1, as well as arrays of single stranded DNA TTAGGG repeats at the ends of human chromosomes [243,244]. POT1 is also found in the telomerase holoenzyme complex, which in addition to POT1 contains TERT, DKC1, WRAP53/TCAB1, NOP10, NHP2, GAR1, TEP1, EST1A, and a telomerase RNA template component (TERC) [245]. Furthermore, POT1 can homodimerize and homo-oligomerize. Human POT1 contains an N-terminal DNA-binding domain (residues 1–300) and a C-terminal protein interaction domain (residues 330–634). Figure 5E shows a crystal structure of a complex between the C-terminal domain of POT1 (POT1C, residues 330–634) and the POT1-binding region of the adrenocortical dysplasia protein homolog (residues 254–336) [212], which is also known as POT1 and TIN2-interacting protein, PIP1, PTOP, TINT1, and TPP1. It is apparent that POT1C forms a bilobal globular structure consisting of an OB-fold and a holiday junction resolvase domain, whereas a POT1-binding motif of TPP1 wraps around POT1C forming a set of loops and disjointed helices involved in extensive interactions with POT1C [212]. This structure of the POT1C-TPP1 complex provides an important illustration of how globular POT1C can bind at least some of its disordered partners.

*Protein mago nashi homolog* (Supplementary Materials Table S2: *UniProt ID P61326*; *190 interactors*; *average disorder content of interactors = 0.33*). MAGOH is a 146 residue-long globular protein found in nucleus, nuclear speckles, and cytoplasm [246]. MAGOH serves as a crucial component of the exon junction complex (EJC) and is also found in the spliceosome C complex [247]. EJC, which plays a major role in posttranscriptional regulation of mRNA, is deposited in a sequence-independent manner at a fixed distance (20–24 nucleotides) upstream of the exon-exon junction of mRNAs and can be involved in the control of alternative splicing [248]. It was pointed out that EJC complex is characterized by a highly dynamic nature, consisting of several core proteins (such as eIF4AIII, Barentsz [Btz], MAGOH, and Y14) and several peripherally associated proteins, such as factors involved in cytoplasmic mRNA surveillance (UPF3 and UPF2), mRNA export factors (REF/Aly, TAP-p15), and splicing coactivators (Srm160, Pinin, RNPS1) [249]. Figure 5F represents a crystal structure of the core EJC complex containing MAGOH (full length), Y14 (residues 66–174), eIF4AIII (full length), Btz (the SELOR domain, residues 137–286), a non-hydrolyzable ATP analog (AMPPNP) and a U_15_ RNA (yellow ribbon) [213]. Similar to POT1C-TPP1 interaction, in the EJC structure, an extended Btz does not have a globular fold, its two separate stretches wrap around both domains of eIF4AIII and also contact MAGOH [213]. In this complex, MAGOH is characterized by a flat antiparallel β-sheet flanked on one side by two parallel α-helices engaged in interaction with the RNA binding domain (RBD) of Y14. Importantly, the β-sheet surface of MAGOH is entirely exposed to solvent and therefore can be engaged in additional PPIs [213].

## 3. Materials and Methods

### 3.1. Dataset and Annotation of Hubs

**Figure 6:**
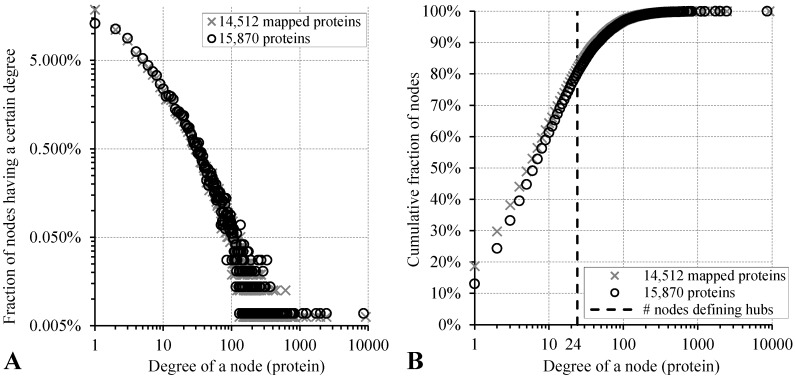
Connectivity of proteins in the human PPI network. Panel (**A**) summarizes the fraction of proteins (nodes in the PPI network) with a given number of PPI interactions (degree). Panel (**B**) gives the cumulative fraction of proteins having a degree less than the corresponding value on the *x*-axis. The circles (crosses) show the results for the original PPI network collected from mentha (the network where proteins were mapped to UniProt). The dashed vertical line in panel (**B**) shows the degree that demarcates hubs, which is defined as the 20% of the most connected nodes.

We collected a set of currently available PPIs for human proteins and used this information to annotate hub proteins and their partners/interactors. The raw data on the PPI were obtained from the *mentha* resource [63]. This resource integrates expert curated PPIs obtained from a comprehensive set of five primary databases: MINT [250], IntAct [251,252], DIP [253], MatrixDB [254], and BioGRID [255]. We extracted all records from taxon 9606 (*Homo sapiens*) for this analysis. These records include 154,395 interactions that cover 15,870 proteins. Next, we mapped these proteins into UniProt [256] to secure their reference sequences and relevant annotations. We were able to map 14,512 proteins that cover 151,461 interactions into the UniProt. The most relevant to our study characteristics of the PPI networks is the degree of the proteins (nodes in the network), which is defined as the number of partners of a given protein. Figure 6 compares the distribution of the degrees between the original set of 15,870 proteins and the set of 14,512 mapped proteins. Figure 6A reveals that the fractions of proteins with a given degree are very similar between the two datasets of proteins. The same observation is true in Figure 6B that compares the two sets of cumulative fractions of proteins with a given degree. This means that the loss of 8.5% of proteins due to mapping into the UniProt, which was necessary to collect the functional and structural annotations for these proteins, did not bias the main characteristics of the underlying network.

As hass already been emphasized (see Introduction section), there are several definitions of hub proteins. We adopted the one recent definition of hubs as the top 20% of proteins with the highest degrees (more most connected proteins) [13]. This definition was also utilized in many other studies [257,258,259]. Figure 6B reveals that the top 20% most connected proteins have degrees ≥24, which is also in close agreement with the hub definition by Aragues et al. [12]. Consequently, the human PPI network includes 2866 hubs, where 13,963 (96.22%) proteins interact with these hubs, 141,346 (93.32%) interactions include at least one hub protein, and 80,557 (53.19%) of these interactions are between hubs. Furthermore, we decided not to sub-divide hubs into subtypes (obligate/party vs. transient/date), since the quality of such categorization was disputed in several works [11,258,260,261]. Instead, we aimed to characterize the entire class of 2866 hub proteins in the context of their already established enrichment in the intrinsic disorder [10,47,48,59,61].

### 3.2. Functional and Structural Characterization of Proteins

We characterized functionally and structurally each protein in the human PPI network. This includes the annotations of the presence of intrinsic disorder, structural stability, evolutionary conservation, ligand binding, catalytic activity, degradation, proteolytic and subcellular targeting sites and annotation of a wide array of post-translational modifications (PTMs).

We comprehensively annotated the intrinsic disorder based on the presence of disordered regions, DPB regions, and MoRF regions. We predicted intrinsic disorder using a consensus of two popular methods: IUPred [144] and ESpritz [145]. We selected these methods based on their favorable predictive quality [262,263,264], the runtime which is sufficiently short to process the 15 thousand proteins, and complementary designs. To the latter point, the consensus includes two versions of IUPred that were designed to specialize in prediction of long disordered regions (30 or more consecutive residues) and short disordered segments (typically present in structured, globular proteins), and three versions of ESpritz that were designed for three types of annotations of disordered residues: using crystal structures, nuclear magnetic resonance structures, and annotations from the DisProt database [265,266]. We implemented the consensus using the majority vote, where three or more out of the five methods must predict the disorder for a given residue to be predicted as disordered. This was motivated by an observation that a consensus secures better predictive quality, when compared to the use of individual predictors [267,268]. The same consensus was utilized in a number of other studies [25,50,66,269,270,271,272]. Similar, consensus-based putative annotations of disorder can be also obtained from the MobiDB [141,142] and D^2^P^2^ [273] databases. In agreement with these works and conventions in this field of research [274], we removed putative disordered segments with less than four consecutive residues. 

We quantified disorder with the disorder content (fraction of disordered residues in a given protein sequence) and the normalized number of long IDPRs (30 or more consecutive residues). We normalized the latter by the unit of length of protein sequence (per 1000 amino acids) to alleviate bias due to the length of the protein chains, i.e., we computed the number of long IDPRs per 1000 amino acids. We considered the long IDPRs since they are recognized as biologically functional disordered domains [24,275,276]. We predicted the DPB regions with the DisoRDPbind method [138,139]. DisoRDPbind was recently shown to outperform other similar methods [138], such as MoRFpred [277], DISOPRED3 [278] and ANCHOR [140]. It is also sufficiently runtime-efficient to process our large dataset. As with all IDPRs, we eliminated putative DPB regions that are shorter than four consecutive residues. We quantified the abundance of these regions with the content of DPB residues (fraction of the DPB residues in a given sequence) and with the normalized number of these regions (number of DPB regions per 1000 amino acids). We also annotated putative MoRF regions, defined as short (between 5 and 25 consecutive residues) DPB regions that undergo disorder-to-order transition upon binding to protein partners. We predicted these regions with a popular and accurate MoRFpred method [264,277]. The same as with the disorder, we assessed the abundance of MoRFs with the content of MoRF residues and with the normalized (per 1000 residues) number of MoRF regions.

On the other end of the protein structure spectrum, we estimated structural stability of these proteins with the number of putative disulfide linkage bonds obtained with the ModPred software [77]. We normalized the number of these bonds per unit of protein chain length (1000 residues). Furthermore, we also assessed other characteristics of proteins in our dataset including the length of their sequences (used as a proxy of protein size) and their evolutionary conservation. We measured the conservation for each residue in an input protein chain using relative entropy [279]:

(1) $$\mathrm{relative}\;\mathrm{entropy}=\overset{N}{\underset{i}{\sum }}p_{i}log_{2}\left(\frac{p_{i}}{p_{ib}}\right)$$

where *N* is the number of amino acid types in the column representing an alignment position in the position specific scoring matrix (PSSM), *p_i_* is the observed frequency of the *i*th amino acid type in the aligned column, and *p_ib_* is the background frequency of the *i*th amino acid found in naturally occurring protein sequences; we computed the latter using the *nr* dataset. PSSM was collected by running the input protein against the *nr* database with the PSI-BLAST algorithm [280] using its default parameters. The relative entropy was empirically shown to be more sensitive to detect functional sites when compared to entropy (which does not utilize *p_ib_* values) and several other conservation measures [279]. This is relevant since functional residues are among the key targets of our analysis. We quantified the conservation per protein and per each putative long IDPR by computing the average of the conservation scores over all residues in a given protein chain and over all residues in a given long disordered segment, respectively.

Next, we annotated and quantified abundance of eukaryotic linear motifs (ELMs) [281], also referred to as short linear motifs (SLiMs). These are short segments of adjacent amino acids that facilitate a wide array of cellular functions, such as protein-ligand interactions, catalytic activity, targeting of subcellular locations, proteolysis, and degradation [281,282,283]. Our focus on these motifs is motivated by an observation that ELMs are often found in IDPRs [284,285]. We extracted ELMs using the ELM resource [282,286,287]. This resource categorizes ELMs into six types: (1) proteolytic cleavage sites (CLV); (2) degradation sites (DEG); (3) posttranslational modification (PTM) sites (MOD); (4) docking sites (DOC) which are associated with the catalytic activity; (5) ligand binding sites (LIG) that mediate non-catalytic protein-ligand interactions; and (6) targeting sites (TRG) that facilitate the subcellular localization of proteins. We quantified abundance of these EML categories by computing the normalized (per 1000 residues) number of corresponding ELMs sites. 

Finally, we annotated and assessed the amount of specific PTM types in our protein set. This analysis extends beyond the analysis based on ELMs that focuses specially on the PTMs associated with SLiMs, by encompassing a broader set of PTMs and quantifying 21 specific PTM types. We annotated the PTMs with the ModPred software [77] that is tuned for eukaryotic organisms. The considered PTM types include acetylation, ADP-ribosylation, amidation, carboxylation, C-linked glycosylation, farnesylation, geranylgeranylation, GPI-anchor amidation, hydroxylation, methylation, myristoylation, N-linked glycosylation, N-terminal acetylation, O-linked glycosylation, palmitoylation, phosphorylation, pupylation, pyrrolidone carboxylic acid, sulfation, sumoylation, and ubiquitination. We computed the overall normalized number of PTMs (across all PTM types) per unit of protein sequence length (1000 residues) where a residue might be counted multiple times if it is annotated to have multiple types of PTMs; the normalized number of residues that have PTMs per 1000 residues where a residue with multiple PTMs is counted just once; and the normalized number of residues with a specific PTM type per 1000 residues.

### 3.3. Annotation of Disease-Linked Proteins

We used the UniProt resource to annotate all diseases that each of the considered 14,512 human proteins is linked with. We mapped the accession numbers of each human protein to the corresponding list of 4296 disease accession numbers. The number of proteins associated with a given disease ranges between a single protein, which occurs for 4085 diseases, and twenty proteins for the non-insulin-dependent diabetes mellitus (accession: DI-02060). Only seven diseases are associated with ten or more proteins. Thus, to ensure sufficient statistical power, we clustered the diseases into broader disease classes. We used the National Library of Medicine’s Medical Subject Headings (MeSH) vocabulary thesaurus to group the 4296 diseases into 26 major classes defined at the second level of the MeSH’s hierarchical structure. The largest number of 4003 proteins are coupled with the MeSH category C16 “congenital, hereditary, and neonatal diseases and abnormalities”, and 18 other classes that we consider in this study have at least 100 associated proteins each. The remaining eight classes include diseases with either a low protein count or that are not related to the human proteins, such as animal and parasitic diseases.

### 3.4. Statistical Analysis

We analyzed whether the considered functional, structural, and disease-association characteristics differ between proteins involved in the hub PPIs (PPIs that involve hubs) that have higher than expected disorder content when compared to proteins associated with the remainder of the hub PPIs. We performed this analysis for both hubs and their interactors. First, we derived the two sets of hubs PPIs; we describe this in Section 3.1. Next, we measured the magnitude and statistical significance of the differences of the considered characteristics between the two sets of proteins. To accomplish that we selected at random 20% of proteins from each of the two sets, quantified a given characteristic for each protein set, and repeated this ten times. This ensures that the measured ten times quantity covers a variety of diverse subpopulations of proteins from a given set. We represented each set of ten measurements with a mean value and we quantified the magnitude of the difference between the two sets of proteins by the relative difference (*Rdiff*) between the two corresponding means defined as:

(2) $$Rdiff=\frac{mean_{disorder\;enriched\;hub\;PPIs}-mean_{remaining\;hub\;PPIs}}{\min\left\{mean_{disorder\;enriched\;hub\;PPIs},\;mean_{remaining\;hub\;PPIs}\right\}}\times 100\text{\%}$$

Positive *Rdiff* values correspond to functional/structural characteristics that are enriched in the hubs (hub interactors) that have higher than expected disorder content in their hub *PPIs* while negative value are for the functional/structural characteristics that are enriched for the remaining hubs (hub interactors). *Rdiff* quantifies the percentage of the increase or decrease, e.g., *Rdiff* = 100% means that the corresponding characteristic is 100% larger in hubs (hub interactors) that have higher than expected disorder content in their hub PPIs compared to the remaining hubs (hub interactors). We also evaluated the statistical significance of the differences between the two sets of ten values. We used the *t*-test if the measurements were normal (we tested normality with the Anderson-Darling test at *p*-value = 0.05); otherwise we used the Wilcoxon test to generate the corresponding *p*-values. We assumed that a given difference is statistically significant if the *p*-value <0.001. Moreover, we assumed that a given functional/structural characteristic is significantly different between the hubs (hub interactors) that have higher than expected disorder content in their hub PPIs compared to the remaining hubs (hub interactors) if the *p*-value <0.001 and |*Rdiff*| is larger than 20%.

## 4. Conclusions

In this study, we analyzed the distribution of intrinsic disorder in the context of hub PPIs for both hubs and their interactors. This type of analysis is different from that of previous studies which were focused solely on the commonness of disorder in the hubs [10,47,48,49,59]. We focused our analysis on hub PPIs pertaining to proteins that have a higher than expected amount of disorder. We quantified and compared a wide range of relevant characteristics of these disorder-enriched hubs and their interactors. The characteristics include structural stability, evolutionary conservation, presence of twenty one types of PTMs, and several categories of functional sites associated with protein binding, catalysis, non-catalytic ligand binding, degradation, proteolytic processing, and subcellular targeting. Our analysis revealed that the disorder-enriched hub PPIs (including both hubs and their interactors) are significantly enriched in the DPB regions and long IDPRs. It also include high levels of catalytic sites, targeting sites, and PTM sites, in particular hydroxylation, phosphorylation, glycosylation, carboxylation, sulfation, and methylation. We also investigated whether disease-associated proteins are common among the proteins involved in the disorder-enriched hub PPIs. Our empirical analysis showed that the hubs registered significant enrichment for 11 out of the considered 18 classes of diseases. They include cancers and diseases of the stomatognathic, endocrine, digestive, respiratory, female urogenital, nervous, and musculoskeletal systems. 

Besides the above analyses that functionally and structurally characterize the overall set of disorder-enriched human hubs PPIs, we also illustrated how specific highly disordered and highly ordered human hubs utilize intrinsic disorder for their promiscuous interactivity. We provided the description of functional and structural characteristics of the five most disordered human hubs, five highly disordered human hubs with the largest number of interactors; and five ordered hubs with the most number of interactors. In agreement with previous studies [61], these analyses revealed that mechanisms by which intrinsic disorder contributes to the binding promiscuity of hub proteins can be grouped in three major categories. The first is where intrinsically disordered hubs interact with multiple partners, both ordered and intrinsically disordered. The second is where the ordered hubs interact with disordered partners that might wrap around hubs. The final category involves hybrid hubs that contain functional domains connected by intrinsically disordered or flexible linkers that facilitate binding diversity.

## Figures and Tables

**Table 1:** Analysis of enrichment in functional and structural characteristics of proteins. We compare hubs/hub interactors/hub interactors that exclude hubs that are associated with the disorder-enriched hub protein-protein interactions (PPIs) that have disorder content higher than expected by over 50% vs. the remaining hubs/hub interactors/hub interactors that exclude hubs. To ensure that results are statistically robust to represent diverse subpopulations of these proteins, we select 20% of proteins at random from each of the two protein sets, quantify a given characteristic for each set, and repeat this ten times. We report the average of the ten repetitions and the relative difference between averages for the two protein sets. We also evaluate the significance of the differences between these measurements. We use the t-test if the data are normal (we test normality with the Anderson-Darling test at *p*-value = 0.05); otherwise we use the Wilcoxon test. Bold font indicates large differences that are statistically significant (*p*-value < 0.001 and |relative difference| ≥ 20%).

Type of Functional/Structural Characteristic ^1^	Measure ^1^	Results for Hubs Involved in Hub PPIs				Results for all Hub Interactors Involved in Hub PPIs				Results for Hub Interactors Involved in Hub PPIs that Exclude Hubs			
		Avg for Disorder-Enriched Proteins	Avg for Remaining Proteins	*p*-Value	Relative Difference	Avg for Disorder-Enriched Proteins	Avg for Remaining Proteins	*p*-Value	Relative Difference	Avg for Disorder-Enriched Proteins	Avg for Remaining Proteins	*p*-Value	Relative Difference
Structural properties	Disorder content	0.542	0.224	<0.001	141%	0.540	0.227	<0.001	138%	0.527	0.213	<0.001	148%
	Number of LIDPRs (per 1000 AAs)	4.428	1.900	<0.001	133%	4.436	1.910	<0.001	132%	4.863	1.954	<0.001	149%
	Number of disulfide links (per 1000 AAs)	0.765	1.240	<0.001	−62%	0.764	1.249	<0.001	−63%	0.973	1.828	<0.001	−47%
Sequence	Average protein chain length	736.8	637.6	<0.001	16%	734.5	653.2	<0.001	12%	791.5	654.2	<0.001	21%
Evolutionary conservation	Average conservation per protein	0.920	1.069	<0.001	−16%	0.930	1.053	<0.001	−13%	0.965	1.048	<0.001	−8%
	Average conservation of LIDPRs	0.735	0.742	0.113	−1%	0.762	0.763	0.811	0%	0.790	0.455	<0.001	74%
Functional regions	MoRF content	0.014	0.008	<0.001	68%	0.014	0.008	<0.001	67%	0.018	0.012	<0.001	53%
	Number of MoRF regions (per 1000 AAs)	1.843	1.090	<0.001	69%	1.834	1.096	<0.001	67%	2.398	1.577	<0.001	52%
	Disordered protein binding (DPB) content	0.230	0.092	<0.001	149%	0.231	0.093	<0.001	148%	0.201	0.074	<0.001	171%
	Number of DPB regions (per 1000 AAs)	8.764	3.665	<0.001	139%	8.784	3.663	<0.001	140%	8.402	3.270	<0.001	157%
Functional motifs (ELMs)	Proteolytic cleavage sites (per 1000 AAs)	0.023	0.033	0.017	−43%	0.022	0.032	0.039	−46%	0.000	0.003	0.044	−89%
	Degradation sites (per 1000 AAs)	0.014	0.019	0.051	−31%	0.014	0.018	0.016	−32%	0.003	0.004	0.342	−40%
	Docking sites for catalysis (per 1000 AAs)	0.137	0.074	<0.001	85%	0.137	0.074	<0.001	85%	0.010	0.005	0.031	88%
	Non-catalytic ligand binding sites (per 1000 AAs)	0.278	0.240	0.026	15%	0.257	0.234	0.061	10%	0.119	0.043	<0.001	174%
	PTM sites (per 1000 AAs)	0.180	0.084	<0.001	114%	0.183	0.087	<0.001	110%	0.028	0.013	0.022	121%
	Targeting sites for localization (per 1000 AAs)	0.149	0.053	<0.001	182%	0.146	0.058	<0.001	151%	0.000	0.008	<0.001	−100%
PTMs	Number of PTMs (per 1000 AAs)	413.3	275.3	<0.001	50%	412.5	275.8	<0.001	50%	410.4	277.9	<0.001	48%
	Number of residues with PTMs (per 1000 AAs)	325.9	226.5	<0.001	44%	325.7	227.1	<0.001	43%	327.3	230.0	<0.001	42%
	Acetylation sites (per 1000 AAs)	9.603	7.954	<0.001	21%	9.481	7.919	<0.001	20%	8.951	7.759	<0.001	15%
	ADP-ribosylation sites (per 1000 AAs)	32.02	18.52	<0.001	73%	31.69	18.39	<0.001	72%	30.97	17.50	<0.001	77%
	Amidation sites (per 1000 AAs)	48.67	36.64	<0.001	33%	49.05	36.59	<0.001	34%	49.62	39.75	<0.001	25%
	Carboxylation sites (per 1000 AAs)	39.24	23.42	<0.001	68%	39.22	23.43	<0.001	67%	38.22	22.28	<0.001	72%
	C-linked_glycosylation sites (per 1000 AAs)	0.200	0.261	<0.001	−31%	0.212	0.287	<0.001	−35%	0.262	0.331	0.004	−21%
	Farnesylation sites (per 1000 AAs)	0.007	0.008	0.303	−21%	0.006	0.008	0.059	−30%	0.008	0.021	0.083	−61%
	Geranylgeranylation sites (per 1000 AAs)	0.008	0.010	0.059	−32%	0.007	0.009	0.045	−40%	0.011	0.031	0.226	−63%
	GPI anchor amidation sites (per 1000 AAs)	1.695	1.621	0.050	5%	1.689	1.626	0.056	4%	1.917	1.865	0.500	3%
	Hydroxylation sites (per 1000 AAs)	26.75	12.56	<0.001	113%	26.98	13.16	<0.001	105%	26.18	12.99	<0.001	101%
	Methylation sites (per 1000 AAs)	15.41	10.27	<0.001	50%	15.33	10.27	<0.001	49%	13.98	9.97	<0.001	40%
	Myristoylation sites (per 1000 AAs)	0.012	0.017	0.002	−39%	0.014	0.013	0.908	1%	0.021	0.015	0.222	41%
	N-linked glycosylation sites (per 1000 AAs)	3.754	3.772	0.577	0%	3.748	3.781	0.356	−1%	3.474	3.660	0.020	−5%
	N-terminal acetylation sites (per 1000 AAs)	0.525	0.517	0.345	2%	0.542	0.515	0.035	5%	0.769	0.797	0.321	−3%
	O-linked glycosylation sites (per 1000 AAs)	14.22	8.18	<0.001	74%	14.71	8.13	<0.001	81%	14.21	7.81	<0.001	82%
	Palmitoylation sites (per 1000 AAs)	1.334	1.734	<0.001	−30%	1.342	1.786	<0.001	−33%	1.783	2.751	<0.001	−35%
	Phosphorylation sites (per 1000 AAs)	44.52	21.63	<0.001	106%	43.75	21.71	<0.001	102%	42.60	18.45	<0.001	131%
	PUPylation sites (per 1000 AAs)	1.949	2.603	<0.001	−34%	1.920	2.633	<0.001	−37%	1.838	2.436	<0.001	−25%
	Pyrrolidone carboxylic acid sites (per 1000 AAs)	4.803	4.357	<0.001	10%	4.813	4.366	<0.001	10%	4.998	4.883	0.197	2%
	Sulfation sites (per 1000 AAs)	3.589	2.291	<0.001	57%	3.603	2.305	<0.001	56%	3.148	2.026	<0.001	55%
	SUMOylation sites (per 1000 AAs)	6.844	6.484	0.003	6%	6.876	6.467	<0.001	6%	6.457	6.682	0.035	−3%
	Ubiquitination sites (per 1000 AAs)	9.377	6.924	<0.001	35%	9.378	6.984	<0.001	34%	9.194	6.931	<0.001	33%

**^1^** Abbreviations: long intrinsically disordered protein region (LIDPR); amino acid (AA); disordered protein binding (DPB); eukaryotic linear motif (ELM); molecular recognition feature (MoRF; short DPB region); posttranslational modification (PTM).

**Table 2:** Analysis of enrichment in the disease associated proteins. We compare the abundance of the disease-linked proteins, measured as the number of the corresponding PPIs that include these proteins that is computed per protein, which are among the hubs and interactors involved in the disorder-enriched hub PPIs vs. their enrichment among PPIs for all human proteins. The 4296 diseases that are associated with human proteins are grouped into disease classes using the MeSH hierarchy. The classes are sorted in descending order by the value of the relative difference between the abundance among the disorder-enriched hub PPIs and all PPIs. To ensure that results are statistically robust to represent diverse subpopulations of these proteins, we select 50% of proteins at random from each of the two protein sets, quantify a given characteristic for each set, and repeat this ten times. We used a larger fraction to define the populations when compared to the results in Table 1 to accommodate the small number of proteins associated with some of the disease classes. We report the average of these repetitions and relative difference between averages for the two protein sets. We also evaluate the significance of the differences between these measurements. We use the *t*-test if the data are normal (we test normality with the Anderson-Darling test at *p*-value = 0.05); otherwise we use the Wilcoxon test.

Disease Class (ID at the Second MeSH Level)		Average for Disorder-Enriched Proteins	Average for all Human Proteins	*p*-Value	Relative Difference
ALL Diseases		0.519	0.433	<0.001	20%
Specific disease classes	Neoplasms, including cancers (C04)	3.150	0.436	<0.001	622%
	Stomatognathic Diseases (C07)	1.833	0.437	<0.001	320%
	Endocrine System Diseases (C19)	1.594	0.432	<0.001	269%
	Digestive System Diseases (C06)	1.440	0.436	<0.001	230%
	Respiratory Tract Diseases (C08)	1.060	0.437	<0.001	143%
	Female Urogenital Diseases and Pregnancy Complications (C13)	0.855	0.437	<0.001	96%
	Nervous System Diseases (C10)	0.775	0.432	<0.001	79%
	Musculoskeletal Diseases (C05)	0.654	0.433	<0.001	51%
	Hemic and Lymphatic Diseases (C15)	0.543	0.434	<0.001	25%
	Pathological Conditions, Signs and Symptoms (C23)	0.467	0.434	<0.001	8%
	Congenital, Hereditary, and Neonatal Diseases and Abnormalities (C16)	0.456	0.433	<0.001	5%
	Male Urogenital Diseases (C12)	0.459	0.436	0.009	5%
	Immune System Diseases (C20)	0.409	0.435	0.81	−6%
	Eye Diseases (C11)	0.373	0.437	<0.001	−17%
	Cardiovascular Diseases (C14)	0.347	0.437	<0.001	−26%
	Nutritional and Metabolic Diseases (C18)	0.248	0.433	<0.001	−75%
	Skin and Connective Tissue Diseases (C17)	0.247	0.435	<0.001	−76%
	Otorhinolaryngologic Diseases (C09)	0.162	0.431	<0.001	−165%

## Supplementary Materials

Supplementary materials can be found at www.mdpi.com/1422-0067/18/12/2761/s1.
